# The Mediating Role of Depression and Pain Catastrophizing in the Relationship between Functional Capacity and Pain Intensity in Patients with Fibromyalgia

**DOI:** 10.1155/2022/9770047

**Published:** 2022-07-16

**Authors:** Casandra I. Montoro, Carmen M. Galvez-Sánchez

**Affiliations:** Department of Psychology, University of Jaén, Spain

## Abstract

**Background:**

Fibromyalgia syndrome (FMS) is a chronic musculoskeletal pain condition characterized by widespread pain, sleep problems (i.e., insomnia and unrefreshing sleep), fatigue, cognitive, and emotional difficulties. Although pain has been proposed the factor mostly impacting in the FMS patients' function, emotional and psychological FMS-associated factors are also known to exert a negative impact in quality of life and functional capacity. Nonetheless, the relationship between these factors and functional limitations in FMS patients is considered to be complex and not clearly defined. Therefore, the present study is aimed at assessing the associations between FMS functional capacity, FMS symptoms (pain, fatigue, insomnia, depression, and state and trait anxiety), and associated psychological factors such as pain catastrophizing, as well as the possible mediating role of these latter in the relationship between pain and FMS functional capacity.

**Method:**

115 women diagnoses with FMS completed a set of self-administered questionnaires to evaluate the clinical and psychological variables of the study.

**Results:**

FMS functional capacity was positively associated with the majority of FMS symptoms except state anxiety. Regression analyses confirmed a greater prediction for FMS functional capacity by depression, fatigue, and pain catastrophizing, in this sequence. Both, pain catastrophizing and depression were important factors mediating the association between clinical pain (total and intensity) and FMS functional capacity.

**Conclusions:**

Findings support a key role of pain catastrophizing and depression in the disability associated to pain in FMS. Treatment goals directed to lessen depression and pain catastrophizing levels should be promoted to reduce the impact of pain in FMS patients' daily function.

## 1. Introduction

Fibromyalgia syndrome (FMS) is a chronic musculoskeletal pain condition characterized by widespread pain, sleep problems (i.e., insomnia and unrefreshing sleep), fatigue, cognitive, and emotional difficulties [1]. The current diagnosis is based on the 2010 American Rheumatology Criteria (ARC). The 2010 ARC criteria, unlike the former criteria, have excluded the tender point count, being more focused on patient-reported somatic symptoms and cognitive difficulties such as memory and attentional impairments [2]. FMS affects between 2.5 and 5% of the worldwide population [1]. The prevalence is 10 times higher in women than in men [1], partially due to a gender bias in the diagnosis [3, 4]. Apart from its high prevalence, FMS entails a high cost for the social and health system, since patients with FMS attend more consultations—both at the level of general medicine and specialized cohort in pain medicine and psychology—and are subjected to more prescriptions and neuroimaging and laboratory tests than the rest of the population [5–9].

FMS is much better understood now than ever before. However, the aetiology remains undetermined. No tissue inflammation or damage explains pain in FMS, but central nervous system (CNS) pain amplification, at least in part, is proposed to be responsible for FMS symptoms [10–12], not only the somatic but also the emotional and cognitive [13–16].

Positive affect disturbances in the context of negative affect [17, 18], aversive emotions [19] and pain catastrophizing [20], also common in FMS, have been associated to the pain modulation impairments [16, 20, 21]. In this context, FMS patients with greater pain catastrophizing levels tend to be less able to distract themselves from pain [20]. Depression and pain catastrophizing have been demonstrated to modulate alterations in central nervous pain processing in FMS [16]. In fact, it has been suggested that FMS patients, in general, tend to selectively attend to information regarding the body and the environment in relation to pain; this phenomenon has been called “cognitive-emotional sensitization” and increase the perception of that pain [22].

Although disabling pain is the hallmark of FMS and the most examined and explanatory factor in relation to functional capacity in FMS patients [23–25], emotional disturbances are also known to reduce functioning in physical, psychological, and social spheres of daily living in FMS patients [26]. For instance, rumination component of pain catastrophizing and depression has been factors directly associated with functional limitations in FMS [23, 27]. Altogether, the aforementioned factors increase the intensity and severity of pain symptoms and cause a great negative impact in functional capacity and quality of life [17, 28–32]. Furthermore, between the clinical FMS symptoms, fatigue has been thought to be one of the most concerning affecting functional's impact of FMS disease [33, 34], even producing intense sedentary behaviours by limiting the ability to carry out daily routines [34, 35].

Despite the last, the relationship between psychological cognitive processes, FMS symptomatology, and functional limitations is considered to be complex [27]. Furthermore, although numerous treatments are available for managing FMS symptoms, the conventional medical therapies that target this pathology produce limited benefits [36]. Regardless the empirical support of the relevance of the emotional and psychological factors in FMS, intervention protocols remain largely pharmacological [36]. Therefore, more comprehensive research analysing the factors mediating the association between pain and FMS functional limitations might be especially important for formulating shared and realistic FMS treatment goals.

Given the aforementioned close relationship between pain and FMS functional capacity as well as between psychological cognitive processes such as pain catastrophizing, affect disturbances (e.g., depression), fatigue and FMS functional capacity, and the necessity of a more comprehensive research in this regards, the present study is aimed at (1) exploring and analysing the association between FMS functional capacity, clinical variables (fatigue, pain, and insomnia), emotional symptoms (depression and anxiety), and psychological cognitive processes (pain catastrophizing); and (2) studying the possible mediating role of these clinical, emotional, and psychological factors on the relationship between pain and FMS functional capacity.

## 2. Materials and Methods

### 2.1. Participants

In total, 115 women with FMS, recruited from the Fibromyalgia Association of Jaén (AFIXA; Spain), participated in this cross-sectional study. All participants were examined by a rheumatologist and met the 2010 American College of Rheumatology criteria for FMS [1]. Exclusion criteria included the presence of metabolic abnormalities, neurological disorders, drug abuse, and severe somatic (e.g., cancer) or psychiatric (e.g., psychotic) diseases.

### 2.2. Instruments and Measures

A semistructured interview was used for obtaining the patients' clinical history and demographic data. The diagnosis of possible mental disorders was assessed by the Structured Clinical Interview for Axis I Disorders of the Diagnostic and Statistical Manual for Mental Disorders (SCID, [37]). In addition, the following self-report questionnaires were administered.

#### 2.2.1. Fibromyalgia Impact Questionnaire (FIQ)

Developed by Burckhardt et al. [38], FIQ is a self-administered questionnaire composed of 10 items that evaluates functional domains affected in FMS patients (e.g., problems with muscle tasks, problems with work, pain, fatigue, stiffness, depression, anxiety, and morning tiredness). The first item of the FIQ (i.e., the physical impairment subscale) is further divided into several sub-items. The maximum possible score of each item is 10. The final score ranges in a continuum between 0 and 100. The lower score indicates greater functional capacity and lower quality of life. In the present study, the Spanish adaptation by Esteve-Vives et al. [39] was used. The internal consistency measured by Cronbach's *α* of the overall FIQ score is 0.82 [39].

#### 2.2.2. McGill Pain Questionnaire (MPQ)

Developed by Melzack [40], MPQ is a 73-item questionnaire that allows for quantification of the pain multidimensional experience. MPQ consists of a ranking of pain descriptors (on an ascending intensity scale) corresponding to the following categories: sensory, miscellanea, affective, and evaluative pain. In the current study, the global MPQ score or total pain (i.e., the sum of the different pain categories and total score between 0 and 167) and the current pain intensity assessed via a 10 cm visual analogue scale (VAS) were used from the Spanish adaptation by Lázaro et al. [41]. Higher score indicates higher levels of pain. The Cronbach's *α* value for total pain is 0.74 [41].

#### 2.2.3. State-Trait Anxiety Inventory (STAI)

Original version is developed by Spielberger et al. [42]. This inventory assesses anxiety in adults differentiating between state anxiety (temporary levels of anxiety) and trait anxiety (long-standing anxiety levels; considered a personality trait). Each condition (i.e., state anxiety and trait anxiety) is measured by 20 items in a 4-point Likert scales. The score ranges between 0 and 60. Higher scores indicate higher levels of anxiety. The Spanish adaptation by Spielberger et al. [43] was applied in the present study. The Cronbach's *α* values are 0.93 for the state anxiety and 0.87 for the trait anxiety scales [43].

#### 2.2.4. Beck Depression Inventory (BDI)

Developed by Beck et al. [44], this 21-item self-reporting scale is applied for assessing the severity of depression symptoms in psychiatric and general populations. Each of the 21 items scores in a 4-point Likert scales ranging from absence of symptoms and severe symptoms. Total score ranges from 0-63. Scores of 20–28 refer to moderate depression. Severe depression is diagnosed with scores of 29-63. Higher score indicates higher severity form of depression. The Spanish adaptation by Vázquez and Sanz [45] was applied. The Cronbach's *α* is 0.95 [45].

#### 2.2.5. Fatigue Severity Scale (FSS)

Developed by Krupp et al. [46], this unidimensional scale measures the impact and severity of fatigue (lack of mental and/or physical energy). FSS is composed by 9 items. Each of the 9 items rates in a 7-point Likert scales. Item-related questions are based on the previous week. Total score ranges between 9 and 63. Higher score indicates higher severity of fatigue. Spanish adaptation by Bulbena et al. [47] was used. The Cronbach's *α* is 0.88 [47].

#### 2.2.6. Oviedo Quality of Sleep Questionnaire (COS)

Developed by Bobes et al. [48], the COS measures the quality of sleep. COS comprises 15 items. Three subscales can be obtained from COS: subjective sleep satisfaction (scoring in a 7-point Likert scales), insomnia, and hypersomnia (both scoring in a 5-point Likert scales). Insomnia subscale of the COS, comprising of 9 items, was used in the study. The insomnia score ranges between 4 and 45. Higher score is indicative of higher insomnia levels. The Cronbach's *α* is 0.88 [48].

#### 2.2.7. Coping Strategies Questionnaire (CSQ)

Original version is developed by Rosenstiel and Keefe [49]. CSQ is a self-administered instrument that includes 39 items in a 6-point Likert scales and assesses the frequency of the use of adaptative and maladaptive pain coping strategies. This instrument was used to evaluate pain catastrophizing. The pain catastrophizing subscale score ranges between 0 and 36. Higher score is indicative of greater tendency to catastrophizing to cope with pain. The Spanish adaptation by Rodriguez et al. [50] was applied. The Cronbach's *α* for pain catastrophizing is 0.89 [50].

### 2.3. Procedure

The G∗Power 3.1.7 program [51] was used within the purpose to stablish the optimal sample size for the correlation and regression analyses. Assuming an alpha level of 0.05, an effect size of 0.50, and a Beta error of 5%, a sample size of 34 participants arose as optimal. The sample size selected was also optimal for the mediation analysis. In single models with one mediator, as in the present, the standard error is accurate for sample sizes of at least 50 [52–54]. The study was conducted in two sessions that took place in the same day. In the first session, a clinical psychologist recorded the sociodemographic data, patients' clinical history, and medication use, assessed the exclusion and inclusion criteria, and performed the SCID interview. During the second session, the questionnaires were fulfilled in a counterbalanced order to avoid the effect of fatigue. Participant details were blinded by a code. The Ethics Committee for Human Research of the University of Jaén approved the study protocol, and all participants provided written informed consent.

### 2.4. Statistical Analysis

First, descriptive analyses were conducted. Pearson correlations between Fibromyalgia Impact Questionnaire (FIQ) score and the clinical and emotional variables measured were computed and tested for significance in an exploratory analysis. Second, multiple regression analyses were performed. Two blocks of variables were entered as predictors in the analyses: (1) age, educational level, and body mass index (simultaneously; enter method) and (2) questionnaire scales, which showed significant correlations with FIQ score in the preceding exploratory analysis (stepwise method). Mediation analysis was performed using the PROCESS macro for SPSS. The mediator variables were determined based on the regression results, and FIQ score was taken as the dependent variable. Moreover, to ensure the sturdiness of the analyses, confidence intervals from bootstrapping estimation techniques were used. For a significant mediation effect, the limits of the confidence interval do not include the 0 value [55, 56]. Mediation analysis fulfilled the assumptions of significant correlations (1) between predictor and dependent variables, (2) between predictor and mediation variables, and (3) between mediation and dependent variables [55, 56]. A total of 5000 bootstrap resamples were used to generate bias-corrected 95% CIs for the indirect effect.

## 3. Results and Discussion

### 3.1. Participants' Demographic and Clinical Data

Table 1 displays the participants' demographic and clinical data.

### 3.2. Associations between Fibromyalgia Impact Questionnaire Scores and the Emotional and Clinical Variables Measured

FIQ scores were positively associated with trait anxiety (*r* = 0.432, *p* ≤ 0.001), depression (*r* = 0.510, *p* ≤ 0.001), fatigue (*r* = 0.315, *p* ≤ 0.001), insomnia (*r* = 0.368, *p* ≤ 0.001), total pain (*r* = 0.280, *p* = 0.002), pain intensity (*r* = 0.372, *p* ≤ 0.001), and pain catastrophizing (*r* = 0.453, *p* ≤ 0.001). No associations were observed for state anxiety (*r* = 0.050, *p* = 0.598), body mass index (*r* = 0.054, *p* = 0.566), age (*r* = 0.152, *p* = 0.105), and educational level (*r* = −0.019, *p* = 0.840).

### 3.3. Results of Multiple Regression Analysis

Significant results of the multiple regression analyses, with respect to the prediction of FIQ score, are presented in Table 2. After controlling for the effects of age, educational level, and body mass index in the first block, depression was the strongest (positive) predictor of FIQ score, explaining the 29% of the variance. Regarding the second regression model, depression followed by fatigue was positively related to FIQ score. Regarding the third regression model, depression, fatigue, and pain catastrophizing (in this sequence) were positively related to FIQ score.

### 3.4. Results of Mediation Analysis

**Figure 1:**
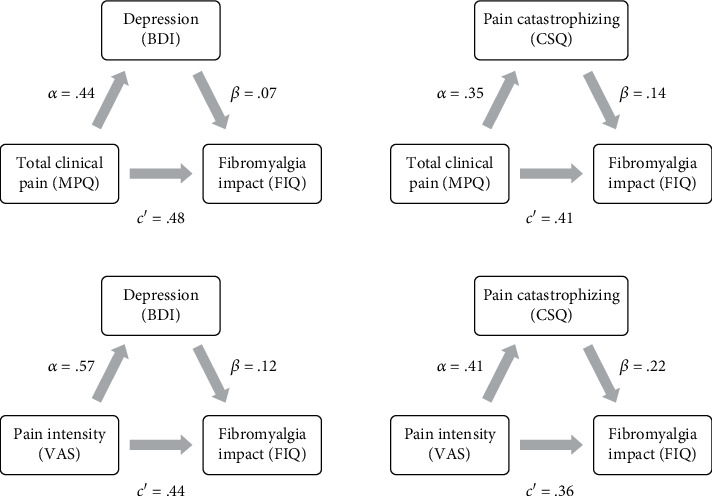
Statistical diagrams of partial mediation effects of depression and pain catastrophizing between clinical pain (total and intensity) and FIQ score. Note: all coefficients are standardized and significant at *p* < 0.01∗. BDI: Beck Depression Inventory; CSQ: Coping Strategies Questionnaire; FIQ: Fibromyalgia Impact Questionnaire; MPQ: McGill Pain Questionnaire; VAS: Visual Analogue Scale.

Depression and pain catastrophizing were significant mediators of the relation between clinical pain (total and intensity) and fibromyalgia capacity (FIQ score). Clinical pain, not only the total but also the intensity (assessed via VAS), increases the levels of depression and pain catastrophizing, provoking a higher negative impact of FMS disease. No mediation effects were found for fatigue (FSS). Further details are provided in Table 3 and Figure 1.

## 4. Discussion

The present study is aimed at comprehensively assessing the association between FMS functional capacity (measured by FIQ) and FMS clinical (fatigue, insomnia and clinical pain) and emotional (anxiety and depression) symptoms and psychological cognitive processes (pain catastrophizing), as well as the possible mediating role of these factors on the association between pain and functional capacity of FMS patients.

FMS patients exhibited similar levels of anxiety, depression, clinical pain, fatigue, insomnia, and pain catastrophizing than previous studies (e.g., [15, 28, 29, 57–61]).

Consistent with our predictions and previous findings, correlation analyses indicated significant positive associations between FMS functional capacity, the FMS symptoms (except state anxiety), and pain catastrophizing (e.g., [23–27, 33, 34]). Depression and anxiety scores have been previously related to higher FIQ score, that is, lower FMS functional capacity (e.g., [62]). The present findings underscore the latter. Though it should not overlook, no significant associations between state anxiety and FMS functional capacity were observed in the present study; which may reflect a specific influence of long-lasting anxiety levels—personality trait—versus temporary anxiety levels in FMS functional capacity.

The mean body mass index in the FMS patients' sample of this study was 28.15 kg/m^2^, which indicates obesity degree in level I [63]; despite the high mean body mass index, this was not related with functional impairments. No significant associations were observed between FMS functional capacity and body mass index. These findings are not in line with previous notions about the contribution of obesity (or elevated body mass index) in FMS severity [64–66], but on the contrary with more recent evidence that does not find a significant association between body mass index and both self-report and performance-based physical functioning [67]. The same occurred with educational level, questioning previous findings revealing a positive impact of education level on the course of the FMS, and considering it as a protective factor for FMS [68].

Regression analyses confirmed a greater prediction for FMS functional capacity by depression, fatigue, and pain catastrophizing, in this sequence. The lack of FMS functional capacity prediction by pain intensity—oppositely to previous studies—may reside in the measuring method used, the MPQ (in the present study) vs. others such as the Brief Pain Inventory (e.g., [69]). Nonetheless, although pain intensity did not account for the prediction of FMS functional capacity as reported in previous research [23–25, 69], the objective of the present study was to explore the mediating impact of clinical, emotional, and psychological factors and also consider to impact on FMS functional capacity, on the well-stablished relation between pain and FMS functional capacity [23–25]. Mediation analysis to this regard has shown that greater levels of pain catastrophizing and depression were significant mediators of the relationship between pain (both pain intensity and total pain) and FMS functional capacity. Similarly, Paschali et al. [69] observed a significant indirect effect of pain catastrophizing on the relationship between pain intensity and FMS functional capacity—also assessed by FIQ revised version.

The tendency to catastrophizing has been proposed to interact with attention-resource allocation and represent a mechanism of chronic pain exacerbation and/or maintenance [20] and may be mediated by preference for fatigue-avoidance goals [34]. Present findings expand this notion. Catastrophizing seems not only exacerbate pain, as proposed by previous research [20], but also intensify the association between pain and FMS functional capacity. It is important note that although there exist studies that confront this assumption; for instance, Lami et al. [70], though found significant associations between FMS disability and pain catastrophizing, not observed a significant mediation effect of pain catastrophizing in the relationship between pain and FMS impact; pain catastrophizing anywise seems to be an important variable contributing to reduced functioning in FMS [69]. To sum up, the findings, from the mediation analysis, support the strong association between negative states (pain catastrophizing and depression) in FMS, the greater intensity and severity of pain symptoms and negative impact on function/quality of life [17, 28–32]; therefore, also the vicious circle that occurs between all these variables.

Considering these results, it is plausible to propose that reducing pain catastrophizing and depression might improve the disability associated to pain in FMS. Treatment goals directed to lessen pain catastrophizing and depression levels should be promoted to reduce the impact of pain in FMS patients' daily function. Indeed, in a recent study, pain catastrophizing has been put forwarded as a potential prognostic factor for rehabilitation associated changes in pain and self-rated physical function (this last in a less extent) in FMS and low back pain [71]. At this regard, acceptance and commitment therapy (ACT), which is considered an effective therapeutic approach for FMS [72, 73], has shown medium-large effect size in the reduction of the FMS impact, measured by the FIQ [74–77]. However, it is important to be cautious with the expected ACT benefits. Lami et al. [70], in an attempt to elucidate the association between pain acceptance and pain, did not find any correlation but a significant influence of pain acceptance on FMS disability. Similar results were observed by Esteve et al. [78].

Regarding depression, it has been proposed to be significantly predicted by pain catastrophizing in patients with persistent pain [79]. Likewise, depression association with pain is suggested to be mediated by pain catastrophizing [70]. So that, the indirect effect of depression in the relation between pain and FMS functional capacity observed in the present study might be likely explained in some part by the associated pain catastrophizing. Notwithstanding, the relevance of emotional factors and coping strategies—supported by present findings—is in line with the increasing transdiagnostic perspective on emotional dysregulation [80]. Assuming depression and pain catastrophizing as part of the transdiagnostic perspective might be important for personalized behaviour management, which is essential for mood regulation as an alternative to pharmacologic treatment in FMS [81]. Most of the studies prompt to include the replacement of maladaptive coping strategies (especially pain catastrophizing) by others more adaptative and mature in the treatment of chronic pain (e.g., [71]); however, our findings go further and encourage a more integrated approach on the management of FMS, not only centred in coping strategies but also in the emotional disturbances—either because of their association with maladaptive coping.

The main limitations of our study pertain to its cross-sectional design which does not account for causal associations, and the no correction for type I errors. Moreover, given the apparent gender bias in the diagnosis of FMS [3, 4], it would be advisable for future research to include males in their samples for making enable gender differences' exploration. In addition, the analyses were based on self-reported measures, which could be highly sensitive to biases such as those related to participant emotional states, in terms of symptom impact and severity [82]. Also, although the relevance of pain catastrophizing and depression in the functional impact of the FMS is clear, possible mediating mechanisms, like the practice of physical exercise or levels of fitness, have been not assessed. Nonetheless, studies to this respect are not clear, even with some of them not showing improvement in FMS functional capacity (measured by FIQ) as a function of fitness and physical exercise accomplishment (e.g., [83]). FMS functional capacity seems to be dependable on the intensity and the kind of the physical exercise. Moderate intensity aerobic aquatic exercise is the one that is suggested to exert greater clinically meaningful improvements in FIQ score [84, 85]. Similarly, studies measuring melatonin secretion as mediating mechanism of depression influence in the relationship between pain and FMS functional capacity are recommended. This recommendation is based on the high demonstrated correlation between the disruption in melatonin secretion and FMS clinical symptoms [86]. Finally, although the sample of patients in this study was sufficient to perform the mediation analysis, future studies with a much larger sample should not be ruled out to increase the effect size for mediation [52].

One strength of this research is to include both clinical and emotional variables; thus, providing a clearer picture of predictors of functional capacity in FMS. In addition, the mediation analysis conducted provides better insight into the complex interrelation between predictors. Finally, the results of the current research have a clear clinical relevance in the development and improvement of FMS treatments.

## 5. Conclusions

In conclusion, findings confirm that the FMS functional impairment is positively related to the majority of FMS symptoms. Among these symptoms, depression, fatigue, and pain catastrophizing (in this sequence) are those with more predictable power. Furthermore, the relevant factors affecting the relationship between pain and FMS functional capacity are pain catastrophizing and depression. Findings support the key role of pain catastrophizing and depression in the disability associated to pain in FMS. Treatment goals directed to lessen depression and pain catastrophizing are strongly recommended to reduce the impact of pain in FMS patients' daily function. The inclusion of these factors in therapy protocols could improve the functional capacity in FMS patients directly and indirectly by the associated reduction in pain perception (intensity and total).

## Figures and Tables

**Table 1:** Mean (*M*) and standard deviation (SD) of participants' demographic and clinical data.

	*n* = 115	
	*M*	SD
Age	51.98	8.23
Body mass index	28.15	2.79
Educational level	9.82	4.00
State anxiety (STAI)	26.55	9.38
Trait anxiety (STAI)	44.09	12.37
Depression (BDI)	33.70	17.10
Fatigue (FSS)	51.12	11.04
Insomnia (COS)	34.91	10.51
Pain intensity (VAS)	5.65	2.51
Total pain (MPQ)	68.70	35.81
Pain catastrophizing (CSQ)	21.63	9.78
Fibromyalgia impact (FIQ)	70.20	16.29

Note. STAI: State-Trait Anxiety Inventory; BDI: Beck Depression Inventory; CSQ: Coping Strategies Questionnaire; FIQ: Fibromyalgia Impact Questionnaire; MPQ: McGill Pain Questionnaire; COS: Oviedo Quality of Sleep Questionnaire; FSS: Fatigue Severity Scale; VAS: Visual Analogue Scale.

**Table 2:** Regression analysis for the prediction of FIQ score by the emotional and clinical variables evaluated.

Dependent variable	Model	Predictor	*β*	*r* ^2^	*t*	*p*
FIQ	1	Depression (BDI)	0.512	0.285	6.33	≤0.001
	2	Depression (BDI)	0.480	0.345	6.12	≤0.001
		Fatigue (FSS)	0.250		3.16	0.002
	3	Depression (BDI)	0.358	0.379	3.88	≤0.001
		Fatigue (FSS)	0.247		3.20	0.0002
		Pain catastrophizing (CSQ)	0.221		2.40	0.018

Note. BDI: Beck Depression Inventory; CSQ: Coping Strategies Questionnaire; FIQ: Fibromyalgia Impact Questionnaire; FSS: Fatigue Severity Scale.

**Table 3:** Results of mediation analysis of the predictors of FIQ score.

Independent variables	Mediator variables	Effect	SE	*p*	Boot LLCI	Boot ULCI
Fibromyalgia impact (FIQ)						
Total clinical pain (MPQ)	Depression (BDI)	0.212	0.052	≤0.001	0.119	0.320
	Pain catastrophizing (CSQ)	0.144	0.045	0.0001	0.066	0.240
Pain intensity (VAS)	Depression (BDI)	0.254	0.062	≤0.001	0.135	0.380
	Pain catastrophizing (CSQ)	0.149	0.042	≤0.001	0.073	0.240

Note: indirect effects are reported. SE: standard error; Boot: bootstrapping results with confidence intervals for the lower (LLCI) and upper limits (ULCI). All coefficients are standardized. BDI: Beck Depression Inventory; CSQ: Coping Strategies Questionnaire; FIQ: Fibromyalgia Impact Questionnaire; MPQ: McGill Pain Questionnaire; VAS: Visual Analogue Scale.

## Data Availability

The data presented in this study are available on request from the corresponding authors.

## References

[1] Wolfe F., Clauw D. J., Fitzcharles M. A. (2011). Fibromyalgia criteria and severity scales for clinical and epidemiological studies: a modification of the ACR preliminary diagnostic criteria for fibromyalgia. *The Journal of Rheumatology*.

[2] Häuser W., Ablin J., Fitzcharles M. A. (2015). Fibromyalgia. *Nature Reviews Disease Primers*.

[3] Srinivasan S., Maloney E., Wright B. (2019). The problematic nature of fibromyalgia diagnosis in the community. *ACR Open Rheumatology*.

[4] Wolfe F., Walitt B., Perrot S., Rasker J. J., Häuser W. (2018). Fibromyalgia diagnosis and biased assessment: sex, prevalence and bias. *PLoS One*.

[5] Busse J. W., Ebrahim S., Connell G. (2013). Systematic review and network meta-analysis of interventions for fibromyalgia: a protocol. *Systematic Reviews*.

[6] Hughes G., Martinez C., Myon E., Taïeb C., Wessely S. (2006). The impact of a diagnosis of fibromyalgia on health care resource use by primary care patients in the UK: an observational study based on clinical practice. *Arthritis and Rheumatism*.

[7] Robinson R. L., Birnbaum H. G., Morley M. A., Sisitsky T., Greenberg P. E., Claxton A. J. (2003). Economic cost and epidemiological characteristics of patients with fibromyalgia claims. *The Journal of Rheumatology*.

[8] Boonen A., van den Heuvel R., van Tubergen A. (2004). Large differences in cost of illness and wellbeing between patients with fibromyalgia, chronic low back pain, or ankylosing spondylitis. *Annals of the Rheumatic Diseases*.

[9] Rivera J., Rejas J., Esteve-Vives J., Vallejo M. A., Sociedad Española del Dolor-SED (2009). Costes económicos asociados al diagnóstico de fibromialgia en España. *Revista de la Sociedad Española del Dolor*.

[10] Brummett C., Clauw D. J. (2018). Fibromyalgia and centralized pain states. *Essentials of Pain Medicine*.

[11] Petersel D. L., Dror V., Cheung R. (2011). Central amplification and fibromyalgia: disorder of pain processing. *Journal of Neuroscience Research*.

[12] Staud R., Nagel S., Robinson M. E., Price D. D. (2009). Enhanced central pain processing of fibromyalgia patients is maintained by muscle afferent input: a randomized, double-blind, placebo-controlled study. *PAIN®*.

[13] Clauw D. J. (2009). Fibromyalgia: an overview. *The American Journal of Medicine*.

[14] Clauw D. J., Arnold L. M., McCarberg B. H. (2011). The science of fibromyalgia. *Mayo Clinic Proceedings*.

[15] Montoro C. I., Duschek S., Ladrón M., de Guevara C., Fernández-Serrano M. J., Reyes del Paso G. A. (2015). Aberrant cerebral blood flow responses during cognition: implications for the understanding of cognitive deficits in fibromyalgia. *Neuropsychology*.

[16] Montoro C. I., Duschek S., Reyes del Paso G. A. (2018). An exploratory analysis of the influence of personality and emotional factors on cerebral blood flow responses during painful stimulation in fibromyalgia. *Scandinavian Journal of Psychology*.

[17] Finan P. H., Zautra A. J., Davis M. C. (2009). Daily affect relations in fibromyalgia patients reveal positive affective disturbance. *Psychosomatic Medicine*.

[18] Malin K., Littlejohn G. O. (2013). Stress modulates key psychological processes and characteristic symptoms in females with fibromyalgia. *Clinical and Experimental Rheumatology*.

[19] Van Middendorp H., Lumley M. A., Jacobs J. W., van Doornen L. J., Bijlsma J. W., Geenen R. (2008). Emotions and emotional approach and avoidance strategies in fibromyalgia. *Journal of Psychosomatic Research*.

[20] Ellingson L. D., Stegner A. J., Schwabacher I. J., Lindheimer J. B., Cook D. B. (2018). Catastrophizing interferes with cognitive modulation of pain in women with fibromyalgia. *Pain Medicine*.

[21] Bhargava J., Hurley J. A. (2021). *Fibromyalgia*.

[22] Brosschot J. F. (2002). Cognitive-emotional sensitization and somatic health complaints. *Scandinavian Journal of Psychology*.

[23] Campos R. P., Vázquez M. I. R. (2012). Health-related quality of life in women with fibromyalgia: clinical and psychological factors associated. *Clinical Rheumatology*.

[24] Carbonell-Baeza A., Aparicio V. A., Sjöström M., Ruiz J. R., Delgado-Fernández M. (2011). Pain and functional capacity in female fibromyalgia patients. *Pain Medicine*.

[25] Mannerkorpi K., Svantesson U., Broberg C. (2006). Relationships between performance-based tests and patients' ratings of activity limitations, self-efficacy, and pain in fibromyalgia. *Archives of Physical Medicine and Rehabilitation*.

[26] Galvez-Sánchez C. M., Duschek S., Del Paso G. A. R. (2019). Psychological impact of fibromyalgia: current perspectives. *Psychology Research and Behavior Management*.

[27] Mahgoub M. Y., Elnady B. M., Abdelkader H. S., Abdelhalem R. A., Hassan W. A. (2020). Comorbidity of fibromyalgia in primary knee osteoarthritis: potential impact on functional status and quality of life. *Open Access Rheumatology: Research and Reviews*.

[28] Galvez-Sánchez C. M., Montoro C. I., Duschek S., Reyes Del Paso G. A. (2020). Depression and trait-anxiety mediate the influence of clinical pain on health- related quality of life in fibromyalgia. *Journal of Affective Disorders*.

[29] Galvez-Sánchez C. M., Montoro C. I., Duschek S., Reyes del Paso G. (2020). Pain catastrophizing mediates the negative influence of pain and trait-anxiety on health-related quality of life in fibromyalgia. *Quality of Life Research: an International Journal of Quality of Life Aspects of Treatment, Care and Rehabilitation*.

[30] Gormsen L., Rosenberg R., Bach F. W., Jensen T. S. (2010). Depression, anxiety, health-related quality of life and pain in patients with chronic fibromyalgia and neuropathic pain. *European Journal of Pain*.

[31] Verbunt J. A., Pernot D. H., Smeets R. J. (2008). Disability and quality of life in patients with fibromyalgia. *Health and Quality of Life Outcomes*.

[32] Zautra A. J., Fasman R., Reich J. W. (2005). Fibromyalgia: evidence for deficits in positive affect regulation. *Psychosomatic Medicine*.

[33] Ojeda B., Dueñas M., Salazar A., Mico J. A., Torres L. M., Failde I. (2018). Factors influencing cognitive impairment in neuropathic and musculoskeletal pain and fibromyalgia. *Pain Medicine*.

[34] Velasco-Furlong L., Gutiérrez-Hermoso L., Mateos-Pintado B. (2020). The 4 U’s rule of fibromyalgia: a proposed model for fatigue in a sample of women with fibromyalgia: a qualitative study. *International Journal of Environmental Research and Public Health*.

[35] Segura-Jiménez V., Borges-Cosic M., Soriano-Maldonado A. (2017). Association of sedentary time and physical activity with pain, fatigue, and impact of fibromyalgia: the al-Ándalus study. *Scandinavian Journal of Medicine & Science in Sports*.

[36] Siracusa R., Paola R. D., Cuzzocrea S., Impellizzeri D. (2021). Fibromyalgia: pathogenesis, mechanisms, diagnosis and treatment options update. *International Journal of Molecular Sciences*.

[37] First M., Spitzer R. L., Gibbon M., Williams J. B. W. (1999). *Entrevista clínica estructurada para los trastornos del eje I del DSM-IV: SCIDI versión clínica*.

[38] Burckhardt C. S., Clark S. R., Bennett R. M. (1991). The fibromyalgia impact questionnaire: development and validation. *The Journal of Rheumatology*.

[39] Esteve-Vives J., Rivera J., Salvat I., de Gracia M., Alegre C. (2007). Proposal for a consensus version of the fibromyalgia impact questionnaire (FIQ) for the Spanish population. *Reumatología Clínica*.

[40] Melzack R. (1975). The McGill pain questionnaire: major properties and scoring methods. *Pain*.

[41] Lázaro C., Bosch F., Torrubia R., Baños J. E. (1994). The development of a Spanish questionnaire for assessing pain: preliminary data concerning reliability and validity. *European Journal of Psychological Assessment*.

[42] Spielberger C. D., Gorsuch R., Lushene R. (1970). *Manual for the State-Trait Anxiety Inventory. Consulting Psychologist*.

[43] Spielberger C. D., Gorsuch R. L., Lushene R. E. (1982). *Manual del Cuestionario de Ansiedad Estado/Rasgo (STAI)*.

[44] Beck A. T., Ward C. H., Mendelson M., Mock J., Erbaugh J. (1961). An inventory for measuring depression. *Archives of General Psychiatry*.

[45] Vázquez C., Sanz J. (1999). Fiabilidad y validez de la versión española del Inventario para la Depresión de Beck de 1978 en pacientes con trastornos psicológicos. *Clinical and Health*.

[46] Krupp L. B., La Rocca N. G., Muir-Nash J., Steinberg A. D. (1989). The fatigue severity scale. *Archives of Neurology*.

[47] Bulbena A., Berrios G. E., Fernández de Larrinoa P. (2000). *Medición clínica en psiquiatría y psicología*.

[48] Bobes J., González M. P., Sáiz P. A., Bascarán M. T., Iglesias C., Fernández J. M. (2000). Propiedades psicométricas del cuestionario Oviedo de Sueño. *Psicothema*.

[49] Rosenstiel A. K., Keefe F. J. (1983). The use of coping strategies in chronic low back pain patients: relationship to patient characteristics and current adjustment. *Pain*.

[50] Rodriguez L., Cano E. J., Blanco A. (2004). Evaluación de las estrategias de afrontamiento de dolor crónico. *Actas Españolas de Psiquiatría*.

[51] Faul F., Erdfelder E., Buchner A., Lang A. G. (2009). Statistical power analyses using G∗power 3.1: tests for correlation and regression analyses. *Behavior Research Methods*.

[52] MacKinnon D. P., Fairchild A. J., Fritz M. S. (2007). Mediation analysis. *Annual Review of Psychology*.

[53] MacKinnon D. P., Lockwood C. M., Hoffman J. M., West S. G., Sheets V. A. (2002). A comparison of methods to test mediation and other intervening variable effects. *Psychological Methods*.

[54] MacKinnon D. P., Warsi G., Dwyer J. H. (1995). A simulation study of mediated effect measures. *Multivariate Behavioral Research*.

[55] Preacher K. J., Hayes A. F. (2004). SPSS and SAS procedures for estimating indirect effects in simple mediation models. *Behavior Research Methods, Instruments, & Computers*.

[56] Preacher K. J., Hayes A. F. (2008). Asymptotic and resampling strategies for assessing and comparing indirect effects in multiple mediator models. *Behavior Research Methods, Instruments, & Computers*.

[57] Bernik M., Sampaio T. P., Gandarela L. (2013). Fibromyalgia comorbid with anxiety disorders and depression: combined medical and psychological treatment. *Current Pain and Headache Reports*.

[58] Castro Sánchez A. M., García López H., Fernández Sánchez M. (2019). Improvement in clinical outcomes after dry needling versus myofascial release on pain pressure thresholds, quality of life, fatigue, pain intensity, quality of sleep, anxiety, and depression in patients with fibromyalgia syndrome. *Disability and Rehabilitation*.

[59] Edwards R. R., Bingham C. O., Bathon J., Haythornthwaite J. A. (2006). Catastrophizing and pain in arthritis, fibromyalgia, and other rheumatic diseases. *Arthritis and Rheumatism*.

[60] Izquierdo-Alventosa R., Inglés M., Cortés-Amador S. (2020). Low-intensity physical exercise improves pain catastrophizing and other psychological and physical aspects in women with fibromyalgia: a randomized controlled trial. *International Journal of Environmental Research and Public Health*.

[61] McCrae C. S., Williams J., Roditi D. (2019). Cognitive behavioral treatments for insomnia and pain in adults with comorbid chronic insomnia and fibromyalgia: clinical outcomes from the SPIN randomized controlled trial. *Sleep*.

[62] Işık-Ulusoy S. (2019). Evaluation of affective temperament and anxiety-depression levels in fibromyalgia patients: a pilot study. *Brazilian Journal of Psychiatry*.

[63] World Health Organization (1998). *Programme of nutrition, family and reproductive health. Obesity. Preventing and managing the global epidemic. Report of a WHO consultation on obesity*.

[64] Okifuji A., Donaldson G. W., Barck L., Fine P. G. (2010). Relationship between fibromyalgia and obesity in pain, function, mood, and sleep. *The Journal of Pain*.

[65] Shapiro J. R., Anderson D. A., Danoff-Burg S. (2005). A pilot study of the effects of behavioral weight loss treatment on fibromyalgia symptoms. *Journal of Psychosomatic Research*.

[66] Koçyiğit B. F., Okyay R. A. (2018). The relationship between body mass index and pain, disease activity, depression and anxiety in women with fibromyalgia. *PeerJ*.

[67] Varallo G., Scarpina F., Giusti E. M. (2021). The role of pain catastrophizing and pain acceptance in performance-based and self-reported physical functioning in individuals with fibromyalgia and obesity. *Journal of Personalized Medicine*.

[68] Demirbag B. C., Bulut A. (2018). Demographic characteristics, clinical findings and functional status in patients with fibromyalgia syndrome. *Diabetes*.

[69] Paschali M., Lazaridou A., Paschalis T., Napadow V., Edwards R. R. (2021). Modifiable psychological factors affecting functioning in fibromyalgia. *Journal of Clinical Medicine*.

[70] Lami M. J., Martínez M. P., Miró E., Sánchez A. I., Guzmán M. A. (2018). Catastrophizing, acceptance, and coping as mediators between pain and emotional distress and disability in fibromyalgia. *Journal of Clinical Psychology in Medical Settings*.

[71] Angst F., Lehmann S., Sandor P. S., Benz T. (2022). Catastrophizing as a prognostic factor for pain and physical function in the multidisciplinary rehabilitation of fibromyalgia and low back pain. *European Journal of Pain (London, England)*.

[72] Ducasse D., Fond G. (2015). Acceptance and commitment therapy. *Encephale*.

[73] Veehof M. M., Oskam M. J., Schreurs K., Bohlmeijer E. T. (2011). Acceptance-based interventions for the treatment of chronic pain: a systematic review and meta-analysis. *Pain*.

[74] Ljótsson B., Atterlöf E., Lagerlöf M. (2014). Internet-delivered acceptance and values-based exposure treatment for fibromyalgia: a pilot study. *Cognitive Behaviour Therapy*.

[75] Luciano J. V., Guallar J. A., Aguado J. (2014). Effectiveness of group acceptance and commitment therapy for fibromyalgia: a 6-month randomized controlled trial (EFFIGACT study). *Pain*.

[76] Simister H. D., Tkachuk G. A., Shay B. L., Vincent N., Pear J. J., Skrabek R. Q. (2018). Randomized controlled trial of online acceptance and commitment therapy for fibromyalgia. *The Journal of Pain*.

[77] Wicksell R. K., Kemani M., Jensen K. (2013). Acceptance and commitment therapy for fibromyalgia: a randomized controlled trial. *European Journal of Pain (London, England)*.

[78] Esteve R., Ramírez-Maestre C., López-Martínez A. E. (2007). Adjustment to chronic pain: the role of pain acceptance, coping strategies, and pain-related cognitions. *Annual of Behavioral Medicine*.

[79] Nicholas M. K., Asghari A. (2006). Investigating acceptance in adjustment to chronic pain: is acceptance broader than we thought?. *Pain*.

[80] Faustino B. (2021). Transdiagnostic perspective on psychological inflexibility and emotional dysregulation. *Behavioural and Cognitive Psychotherapy*.

[81] Davydov D. M., Galvez-Sánchez C. M., Montoro C. I., de Guevara C. M. L., Reyes del Paso G. A. (2021). Personalized behavior management as a replacement for medications for pain control and mood regulation. *Scientific Reports*.

[82] Watson D., Pennebaker J. W. (1989). Health complaints, stress, and distress: exploring the central role of negative affectivity. *Psychological Review*.

[83] Valim V., Oliveira L., Suda A. (2003). Aerobic fitness effects in fibromyalgia. *The Journal of Rheumatology*.

[84] Aborde J., Barrientos A. R., Nacef H. B. (1961). For persons with fibromyalgia, does aquatic exercise produce clinically meaningful changes in FIQ/VAS scores?. *The Journal of Aquatic Physical Therapy*.

[85] Beck A. T., Ward C. H., Mendelson M., Mock J., Erbaugh J. (1961). Inventory for measuring depression. *Archives of General Psychiatry*.

[86] Caumo W., Hidalgo M. P., Souza A., Torres I. L., Antunes L. C. (2019). Melatonin is a biomarker of circadian dysregulation and is correlated with major depression and fibromyalgia symptom severity. *Journal of Pain Research*.

